# COVID-19 and atrial fibrillation: Intercepting lines

**DOI:** 10.3389/fcvm.2023.1093053

**Published:** 2023-01-23

**Authors:** Maria Donniacuo, Antonella De Angelis, Concetta Rafaniello, Eleonora Cianflone, Pasquale Paolisso, Daniele Torella, Gerolamo Sibilio, Giuseppe Paolisso, Giuseppe Castaldo, Konrad Urbanek, Francesco Rossi, Liberato Berrino, Donato Cappetta

**Affiliations:** ^1^Department of Experimental Medicine, University of Campania “Luigi Vanvitelli”, Naples, Italy; ^2^Department of Medical and Surgical Sciences, Magna Græcia University, Catanzaro, Italy; ^3^Cardiovascular Center Aalst, OLV Hospital, Aalst, Belgium; ^4^Department of Advanced Biomedical Sciences, University of Naples “Federico II”, Naples, Italy; ^5^Department of Experimental and Clinical Medicine, Magna Græcia University, Catanzaro, Italy; ^6^Santa Maria delle Grazie Hospital, Pozzuoli, Italy; ^7^Department of Advanced Medical and Surgical Sciences, University of Campania “Luigi Vanvitelli”, Naples, Italy; ^8^Department of Molecular Medicine and Medical Biotechnology, University of Naples “Federico II”, Naples, Italy; ^9^CEINGE Advanced Biotechnologies, Naples, Italy; ^10^Department of Biological and Environmental Sciences and Technologies, University of Salento, Lecce, Italy

**Keywords:** COVID-19, inflammation, atrial fibrillation, COVID-19 drugs, atrial remodeling

## Abstract

Almost 20% of COVID-19 patients have a history of atrial fibrillation (AF), but also a new-onset AF represents a frequent complication in COVID-19. Clinical evidence demonstrates that COVID-19, by promoting the evolution of a prothrombotic state, increases the susceptibility to arrhythmic events during the infective stages and presumably during post-recovery. AF itself is the most frequent form of arrhythmia and is associated with substantial morbidity and mortality. One of the molecular factors involved in COVID-19-related AF episodes is the angiotensin-converting enzyme (ACE) 2 availability. Severe acute respiratory syndrome coronavirus 2 (SARS-CoV-2) uses ACE2 to enter and infect multiple cells. Atrial ACE2 internalization after binding to SARS-CoV-2 results in a raise of angiotensin (Ang) II, and in a suppression of cardioprotective Ang(1–7) formation, and thereby promoting cardiac hypertrophy, fibrosis and oxidative stress. Furthermore, several pharmacological agents used in COVID-19 patients may have a higher risk of inducing electrophysiological changes and cardiac dysfunction. Azithromycin, lopinavir/ritonavir, ibrutinib, and remdesivir, used in the treatment of COVID-19, may predispose to an increased risk of cardiac arrhythmia. In this review, putative mechanisms involved in COVID-19-related AF episodes and the cardiovascular safety profile of drugs used for the treatment of COVID-19 are summarized.

## 1. Introduction

An outburst of pneumonia caused by severe acute respiratory syndrome coronavirus 2 (SARS-CoV-2) was reported in December 2019, prompting Health Agencies to issue a public health emergency (1). Since then, the viral infection has reached epidemic proportions, affecting nearly 300 million people in 2 years (2). Most cases are asymptomatic or associated with mild symptoms, but a significant minority of patients develops severe symptoms that can result in multiple organ failure and death (3). Mortality rate is increased by age, pre-existing cardiovascular diseases and metabolic disorders conditions such as hypertension, heart failure, type 2 diabetes and obesity (4–6). The main extra-pulmonary site involved in COVID-19 is the cardiovascular system. Early cardiac injury, evidenced by elevated cardiac biomarkers, is associated to mortality, and has been reported in hospitalized COVID-19 patients (7).

**FIGURE 1:**
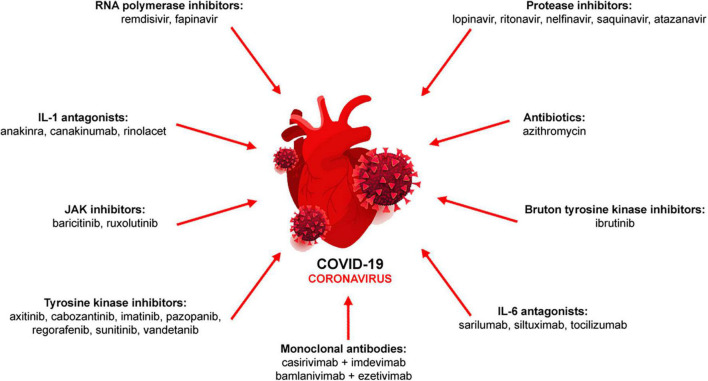
Pharmacological therapy of COVID-19. Antiviral (RNA polymerase and protease inhibitors, monoclonal antibodies), immunomodulatory (IL-1 and IL-6 antagonists, JAK inhibitors) or other drugs (antibiotics, tyrosine kinase inhibitors) are used either alone or in combination.

Due to the initial lack of knowledge of COVID-19 pathophysiology and thus, effective treatments, compassionate and emergency use of drugs including numerous antibodies have been approved. During the pandemic phase, several drug classes have been used either as monotherapy or in combination to minimize disease severity (8–10; Figure 1).

Cardiac manifestations related to COVID-19 infection include arrhythmias, acute myocardial infarction and myocarditis (11). While the etiology of cardiac manifestations is multifactorial, it is possible that also genetic background, such as clinically silent and previously unrecognized channelopathies, can make these patients susceptible to cardiac arrhythmias. Interestingly, atrial fibrillation (AF) and COVID-19 infection appear to share some pathophysiological features, both being driven by an immune response, with inflammatory markers, such as C-reactive protein and cytokine interleukin (IL)-6, correlating with disease severity and mortality (12–14). Therefore, it is understandable that a high incidence of AF with COVID-19 has been reported (15–17).

Although a common immunoinflammatory substrate between COVID-19 and AF has emerged, a few studies have evaluate whether the COVID-19 inflammatory mediators are uniquely responsible for AF or whether this arrhythmia is related to a non-specific product of severe viral respiratory illness.

## 2. The outline of AF pathophysiology

Atrial fibrillation (AF), the most common type of cardiac arrhythmia, is an evolving age-related disease where co-morbidities or lifestyle conditions, such as hypertension, diabetes mellitus, obesity, chronic kidney disease and inflammatory diseases, play a pivotal role (18, 19). In 30% of cases, however, the arrhythmia manifests in asymptomatic subjects not affected by any of the previous pathologies, significantly reducing the quality of life (20).

Atrial fibrillation (AF) pathogenesis is associated with atrial electrical and structural remodeling (21, 22). Short and long-term electrical remodeling present different substrates: the former is related to an altered intracellular Ca^2+^ level *via* ryanodine receptors (RyRs) and voltage-dependent L-type Ca^2+^ current inactivation, the latter is related to reduce levels of mRNA transcript encoding ion channels or to post-transcriptional mechanisms (23). From the structural viewpoint, pro-fibrotic atrial remodeling has been shown to increase the AF susceptibility, contributing to the transition from paroxysmal to persistent or permanent AF (24). Myocardial fibrosis and the activation of its molecular and cellular drivers have been observed in atrial tissue of AF patients, demonstrating also a positive correlation between the degree of atrial fibrosis and the persistence of AF (25). Of note, pharmacotherapy with anti-fibrotic potential (i.e., statins and renin-angiotensin-aldosterone system (RAAS) inhibitors) effectively limits the formation of this structural substrate of AF (26).

Further, inflammation and reactive oxygen species also contribute to unbalanced homeostasis of atrial myocardium, promoting not only the onset but also the AF duration. Inflammatory cell infiltration and increased serum levels of inflammatory mediators, such as tumor necrosis factor-α (TNF-α), interleukin (IL)-1β, IL-6, IL-8, and IL-10, have been found in AF patients, correlating with AF duration and severity (27). Accordingly, treatments pointing at decreasing inflammatory response and oxidative stress have shown promising results by alleviating atrial structural and electrical remodeling (28).

Undoubtedly, a deeper understanding of the underlying AF pathophysiology as well as the individual patient characteristics are still needed to expand the effective and safe therapeutic armamentarium, and optimize a personalized pharmacotherapy.

### 2.1. AF in COVID-19 patients

Atrial fibrillation (AF) is the most common form of arrhythmia in COVID-19 patients, and can be the first sign even prior to evident respiratory distress (29). Almost 20% of COVID-19 patients have a history of AF, but also a new-onset AF represents a frequent complication in COVID-19 with a risk ranging between 10 and 18% (30–32). In a multicenter retrospective cohort study, the incidence of AF during hospitalization is 10% and the incidence of new-onset AF in patients without a pre-existing history of atrial arrhythmias is 4% (33).

Clinical evidence from hospitalized patients has demonstrated that COVID-19, by promoting the evolution toward a prothrombotic state, increases the susceptibility to AF during the infective stages and presumably during post-recovery (34). Meta-analysis studies revealed that pre-existing AF in patients with COVID-19 is associated with increased in-hospital mortality, post-discharge mortality and mechanical ventilation use (35). New-onset AF in the context of COVID-19-related pneumonia is linked to adverse prognosis, suggesting a correlation with the degree of inflammatory and hypoxemic viral insult that increase the hypercoagulable state, endothelial dysfunction, and oxidative stress (36). In general, as for all critically ill patients in which AF independently increases the risk of stroke, length of hospitalization, and death (37), this arrhythmia complicates the clinical course also in COVID-19 patients.

### 2.2. AF and COVID-19: Mechanistic insights

**FIGURE 2:**
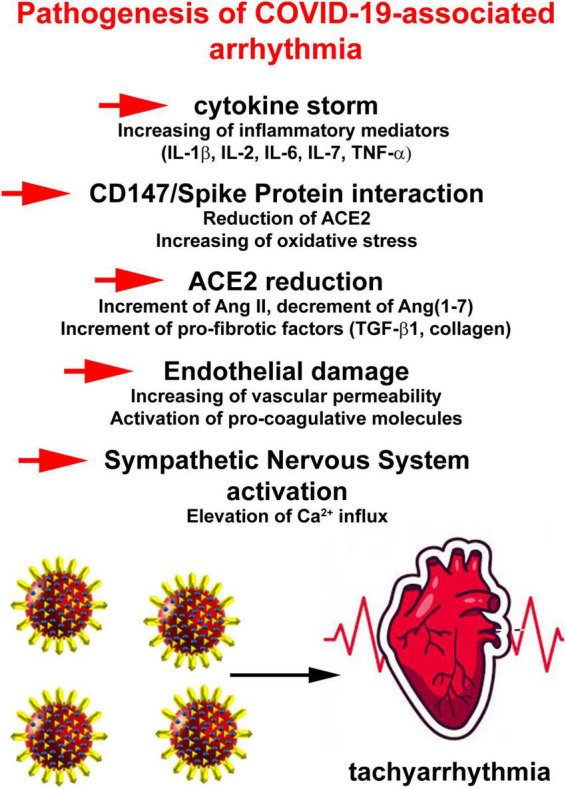
Pathophysiology of COVID-19-related atrial fibrillation (AF) events. Putative mechanisms include a reduced availability of angiotensin-converting enzyme 2, binding of viral spike protein to CD147 or sialic acid, enhancement of inflammatory signaling culminating in cytokine storm, endothelial damage, and increased adrenergic drive.

In attempt to outline a pathophysiology of COVID-19-related AF, several putative mechanisms have been proposed. They include a reduced availability of angiotensin-converting enzyme (ACE) 2, binding of viral spike protein to CD147 or sialic acid, enhancement of inflammatory signaling culminating in cytokine storm, endothelial damage and increased adrenergic drive (38; Figure 2).

Angiotensin-converting enzyme 2 (ACE2) converts angiotensin (Ang) I and Ang II into active peptides Ang(1–9) and Ang(1–7), respectively, which provide counter-regulatory effects for the classical RAAS axis (39, 40). After the cleavage of the viral spike protein, SARS-CoV-2 uses ACE2 to enter and infect host cells such as cardiomyocytes, pericytes, pneumocytes, endothelial cells, and macrophages (41–43). This interaction results in a reduction of ACE2 on the cell surface, suppressing a key pathway for the degradation of Ang II to form cardioprotective Ang(1–7). The consequent increase in Ang II/Ang(1–7) ratio shifts the balance to Ang II thereby promoting cardiac hypertrophy, vasoconstriction, tissue fibrosis, and oxidative stress (9). Moreover, atrial ACE2 catabolizes transforming growth factor-β1 (TGF-β1), the principal pro-fibrotic cytokine (44). This may underlie atrial arrhythmogenesis and potentially increase the susceptibility to AF in COVID-19 patients (45). ACE2 is also involved in the regulation of the cardiac action potential. Ang(1–7) modulates Ca^2+^ homeostasis and cellular electrophysiology in atrial tissue and pulmonary veins (46, 47). Experimental animal studies have demonstrated an increased expression of ACE2 receptor following treatment with ACE inhibitors and angiotensin receptor blockers (48). Studies on ACE2 expression conducted in experimental models and human transcriptome to identify the organs more susceptible to this infection have revealed a low level in the lung, mainly limited to a small fraction of type II alveolar epithelial cells (49). It has been hypothesized that the massive release of inflammatory cytokines is responsible of an increase in ACE2 expression, thus potentiating the infection (50–52).

CD147 is an adjunctive player that facilitates SARS-CoV-2 invasion into host cells, including cardiomyocytes, by interacting with viral spike protein (53, 54). Although the involvement of CD147 in SARS-CoV-2 infection is still debated, it may represent a possible therapeutic target to challenge COVID-19 (55, 56). Furthermore, CD147 upregulates cytokine expression, stimulates oxidative stress in cardiomyocytes and promotes negative ionotropic effects (57). In cardiomyocytes, CD147 is a strong inducer of IL-18 that activates matrix metalloproteinases (MMPs) and circulating IL-18 levels positively correlates with AF development (58). MMP-9 increases extracellular matrix components degradation but can also activate TGF-β1, favoring myocardial adverse remodeling. Higher plasma levels of MMP-9 found in AF patients suggest that MMP-9 can be a marker of atrial remodeling (59). Interestingly, also in COVID-19 patients increased circulating MMP-9 is found (60). Although the role of this protease in tissue damage and repair at the pulmonary level remains to be clarified, the emerging picture is that the levels of MMP-9 increases during the course of the disease and correlates with the number of circulating inflammatory cells (61, 62).

The spike proteins of several coronaviruses bind to sialic acid on the cell surface (63). N-acetylneuraminic acid, the predominant sialic acid in human glycoproteins and gangliosides. By activating RhoA signaling, N-acetylneuraminic acid may trigger cardiac fibrosis and atrial enlargement, contributing to AF pathophysiology (64, 65).

Another promising marker involved in extracellular matrix formation is galectin-3 that plays a role in the progression of atrial fibrosis. It is expressed in fibroblasts, activated macrophages, neutrophils and mast cells and participates in several processes involved in fibrogenesis. In AF, elevated levels of galectin-3 correlate with advanced disease and worse outcomes (66). Notably, galectin-3 levels are increased in serum of COVID-19 patients and correlates with COVID-19 severity (67, 68). Increased levels of aldosterone, another key player in adverse myocardial remodeling, are also found (67). A distinctive hallmark of SARS-CoV-2 infection is systemic immune cell over-activation, with an imbalance between T–helper–1 (Th1) and Th2 cells, elevated levels of IL-1β, IL-2, IL-6, IL-7, interferons, TNF-α, monocyte chemoattractant protein-1 and macrophage inflammatory protein-1A among others (69–72). At the cardiac level, pro-inflammatory cytokines, in particular IL-6, stimulates vascular smooth muscle proliferation, endothelial cell and platelets activation, and leads to apoptosis or necrosis of myocardial cells, which may mediate intra-atrial repolarization and conduction disturbances (73). Raised levels of IL-6 in COVID-19 deaths suggest that virus-driven hyper-inflammation is strictly correlated to and increased susceptibility to lethal arrhythmia (74). SARS-CoV-2, through its binding to ACE2, purinergic receptors and components of the complement-mediated pathway, also stimulates the formation of the Nod-like receptor pyrin domains-containing 3 (NLRP3) inflammasome (75, 76). The NLRP3 inflammasome, in turn, triggers an immune response that leads to a further release of pro-inflammatory cytokines, inflammatory cell death, and Ang II-mediated tissue remodeling (77). There is a causal link between activation of the NLRP3 inflammasome in atrial cardiomyocytes and AF development. The mechanisms underlying the pro-arrhythmic effects of NLRP3 inflammasome take account of abnormal diastolic RyR2-mediated sarcoplasmic reticulum Ca^2+^ release with generation of pro-arrhythmic delayed afterdepolarizations (DADs), continued activation of ultra-rapid delayed rectifier K^+^ current with action potential abbreviation, and atrial hypertrophy and fibrosis (27, 38, 78).

Another relevant pathophysiological component in patients with severe COVID-19 is endothelial dysfunction that may be related, in addition and in combination to cytokine network, to progression and worsening of AF episodes (79–81). The mechanisms are various and not completely understood. It has been hypothesized that a downregulation of ACE2 activates the kallikrein-bradykinin system, increasing vascular permeability to immune cells, which upon activated, produce reactive oxygen species, cytokine and vasoactive molecules release, which lead to endothelial cell dysfunction and loss (82, 83). Impairment of endothelium compartment by SARS-CoV-2, by amplifying the expression of pro-coagulative molecules (i.e., tissue factor) and reducing the level of endothelial antithrombotic molecules, may be responsible for an enhancement of the coagulation cascade (84).

Lastly, in COVID-19 as well as in other viral infection, the activation of sympathetic nervous system takes place (85, 86). The mechanisms linking the increase in the sympathetic tone to AF episodes can involve increase in Ca^2+^ influx and overload in cardiomyocytes. This elevates the frequency of spontaneous diastolic Ca^2+^ release *via* RyR with subsequent generation of DADs and action potentials, which increase the probability of AF events (87).

Overall, there are several common pathophysiological points between COVID-19 and AF, and a potential mechanistic link emerges as a valid working hypothesis. Additionally, pre-existing genetic background consisting of ion-channel and gap junctional protein abnormalities may form the molecular substrate that favors the abnormal conduction properties and electrical activity in the atrial myocardium.

### 2.3. AF management in COVID-19 patients

Deregulation of the coagulation system and the risk of thromboembolism are highly relevant for both AF and COVID-19. Pre-existing antithrombotic therapy may be associated with lower odds of COVID-19 death (88, 89). Although no specific therapy has been recommended, anticoagulant therapy is required. Systemic anticoagulants have been reported to reduce mortality in hospitalized patients with COVID-19 and symptoms of coagulation disorders (90). The use of non-vitamin K antagonist oral anticoagulants (NOACs) in hospitalized COVID-19 patients with AF is a therapeutic alternative (91).

However, clinical findings have demonstrated relevant interactions between COVID-19 drugs and anticoagulants. In particular, lopinavir/ritonavir, *via* cytochrome P450 CYP3A4, may increase the bleeding risk, and NOACs should be avoided (92). As heparins are not expected to interact, they may be considered a safe option. In addition to the antithrombotic effect, heparin anti-inflammatory actions are relevant in this setting (93).

There are few data on the efficacy of rhythm and rate control in patients with AF and COVID-19, and combination therapy with antiarrhythmics and anticoagulants is associated with substantial side effects (94). Cardioversion should be considered in patients with hemodynamic instability, and intravenous amiodarone is the antiarrhythmic drug of choice for rhythm control (95). Rate control may be achieved by intravenous diltiazem (96). In stable patients on antiviral treatment, the interruption of antiarrhythmic drugs is preferable while the initiation of rate control therapy with β-blockers or non-dihydropyridine calcium channel blockers allows the use of antiviral drugs without risk of prolongation of the QT interval (97). Generally, drug-drug interactions should be considered before starting therapy.

To date, it is not clear if AF events experienced by COVID-19 patients are transitory phenomena or they progress into permanent AF. Nonetheless, it remains critical to direct a strict focus to adverse effects of COVID-19 and plan specific screening for irregular heartbeats. To avoid complications, an accurate diagnosis of AF is crucial and remains a major challenge.

A randomized trial recruiting more than 1,000 patients with confirmed COVID-19 has demonstrated that therapeutic-dose anticoagulation does not affect the probability of survival to hospital discharge (98). Therefore, assessing the risk for anticoagulation measures by lowering therapeutic-dose anticoagulation in COVID-19 patients at high risk of AF is a strategy that is worth investigating, although it must be taken into account that AF, without adequate treatment, leads to serious complications.

### 2.4. Risk of drug-related cardiac arrhythmias during COVID-19 therapy

Pharmacological agents commonly used in COVID-19 patients may have a risk of inducing electrophysiological changes and severe and potentially fatal cardiac dysfunction, such as torsades de pointes, ventricular tachycardia and fibrillation (99). Furthermore, patients with underlying heart disease such as inherited arrhythmia syndromes (long QT or Brugada syndromes) are predisposed to an increased risk of cardiac arrhythmias (100). The sum of pharmacotherapy and hereditary factors represents a hazardous combination of pro-arrhythmogenic effects. The use of lopinavir/ritonavir combination has been associated with and increased QT prolongation through a multichannel blocking properties (101). Ibrutinib, the first human Bruton’s tyrosine kinase inhibitor (TKI), has been largely studied in hospitalized COVID-19 patients due to its potential to lessen lung inflammation and injury (102, 103). However, clinical data have revealed an increased risk of atrial and ventricular arrhythmias, sinoatrial arrest, and heart failure; therefore, patients on ibrutinib therapy must be carefully monitored (104). The postulated mechanism seems to be a disrupted Ca^2+^ handling in the myocardium, favoring DADs. Other TKIs exert *in vitro* inhibitory activity against SARS-CoV-2 (105), although patients treated with TKIs have experienced cardiac toxicity (106). This pro-arrhytmogenic effect may be related to a modulation of ionic channels (decreasing K^+^ current amplitude, and interfering with Na^+^ and Ca^2+^ currents).

Remdesivir is an antiviral drugs initially used in patients infected by Ebola virus, and authorized for treatment of COVID-19 disease in hospitalized patients (107). Few studies on remdesivir have pointed out its adverse effects on the cardiovascular system. COVID-19 patients with an oxygen saturation of less than 94% receiving intravenous remdesivir have experienced AF and adverse events are more prevalent in patients undergoing invasive ventilation. However, the interpretation is inconclusive due to small sample size, short follow-up and absence of a control group (108). In another study, elevated plasma concentration of remdesivir following intravenous administration significantly increased the risk of QT prolongation and torsades de pointes (109). The analysis of EudraVigilance database has revealed a two-fold increased risk of an adverse cardiac event associated with remdesivir in comparison with hydroxychloroquine and azithromycin. Cardiac arrhythmias are the most reported events (110). Additional evidence has shown that remdesivir, for its chemical structure of adenosine nucleotide analog and pharmacological profile, may act as a blocker of the atrioventricular node and be pro-arrhythmic especially in patients with structural heart disease (111). Thus, the use of other drugs and a pre-existing risk have to be considered to establish cardiovascular risk for patients with COVID-19 qualified for remdesivir treatment.

## 3. Conclusion

Although most of the symptoms in COVID-19 patients involve the respiratory system, a significant fraction of patients presents serious cardiovascular complications. Arrhythmias are one of the main cardiac manifestations of COVID-19 with AF being the most common form of arrhythmia in these patients. While the pathophysiology underlying AF onset in COVID-19 patients are incompletely understood, from the clinical and basic research emerges an array of common mechanisms in the onset of AF and COVID-19 development. A direct viral invasion of myocardial cells, systemic inflammation with the release of inflammatory cytokines and pro-fibrotic mediators, along with changes in ion channel physiology and local RAAS, have been noted.

Pharmacological agents commonly used in COVID-19 patients may carry a risk of inducing electrophysiological changes and the management of arrhythmias should be based on evidence-based guidelines, with consideration of severity of COVID-19, the nature of AF and the concomitant use of antimicrobial and anti-inflammatory drugs. Finally, “off label” or “new” drugs used in acute COVID-19, vaccines with favorable efficacy and safety profile, and residual risk related to the “long COVID syndrome,” need attention also in the context of arrhythmic manifestations.

## Author contributions

MD, KU, FR, and DC: conceptualization and writing. MD, CR, EC, and DC: literature collection and visualization. AD, PP, DT, GS, GP, GC, KU, LB, and DC: review and editing. All authors contributed to the article and approved the submitted version.

## References

[1] Jee Y. WHO international health regulations emergency committee for the COVID-19 outbreak. *Epidemiol Health.* (2020) 42:E2020013. 10.4178/epih.e2020013 32192278 PMC7285442

[2] WHO. *The World Health Organization COVID-19 Dashboard Provides Up-to-Date Epidemiological Data About the COVID-19 Pandemic.* Geneva: WHO (2020).

[3] Mohanty S Satapathy A Naidu M Mukhopadhyay S Sharma S Barton L Severe acute respiratory syndrome coronavirus-2 (SARS-CoV-2) and coronavirus disease 19 (COVID-19)–anatomic pathology perspective on current knowledge. *Diagn Pathol.* (2020) 15:103. 10.1186/s13000-020-01017-8 32799894 PMC7427697

[4] Wu Z McGoogan J. Characteristics of and important lessons from the coronavirus disease 2019 (COVID-19) outbreak in china: summary of a report of 72314 cases from the Chinese center for disease control and prevention. *JAMA.* (2020) 323:1239–42. 10.1001/jama.2020.2648 32091533

[5] Zhou F Yu T Du R Fan G Liu Y Liu Z Clinical course and risk factors for mortality of adult inpatients with COVID-19 in Wuhan, China: a retrospective cohort study. *Lancet.* (2020) 395:1054–62. 10.1016/S0140-6736(20)30566-3 32171076 PMC7270627

[6] Martínez-Rubio A Ascoeta S Taibi F Soldevila J. Coronavirus disease 2019 and cardiac arrhythmias. *Eur Cardiol.* (2020) 15:e66. 10.15420/ecr.2020.23 33294034 PMC7689870

[7] Shi S Qin M Shen B Cai Y Liu T Yang F Association of cardiac injury with mortality in hospitalized patients with COVID-19 in Wuhan, China. *JAMA Cardiol.* (2020) 5:802–10. 10.1001/jamacardio.2020.0950 32211816 PMC7097841

[8] Scavone C Brusco S Bertini M Sportiello L Rafaniello C Zoccoli A Current pharmacological treatments for COVID-19: what’s next? *Br J Pharmacol.* (2020) 177:4813–24. 10.1111/bph.15072 32329520 PMC7264618

[9] Mascolo A Scavone C Rafaniello C De Angelis A Urbanek K di Mauro G The role of renin-angiotensin-aldosterone system in the heart and lung: focus on COVID-19. *Front Pharmacol.* (2021) 12:667254. 10.3389/fphar.2021.667254 33959029 PMC8093861

[10] Scavone C Mascolo A Rafaniello C Sportiello L Trama U Zoccoli A Therapeutic strategies to fight COVID-19: which is the status artis? *Br J Pharmacol.* (2022) 179:2128–48. 10.1111/bph.15452 33960398 PMC8239658

[11] Gopinathannair R Merchant F Lakkireddy D Etheridge S Feigofsky S Han J COVID-19 and cardiac arrhythmias: a global perspective on arrhythmia characteristics and management strategies. *J Interv Card Electrophysiol.* (2020) 59:329–36. 10.1007/s10840-020-00789-9 32494896 PMC7268965

[12] Liu F Li L Xu M Wu J Luo D Zhu Y Prognostic value of interleukin-6, C-reactive protein, and procalcitonin in patients with COVID-19. *J Clin Virol.* (2020) 127:104370. 10.1016/j.jcv.2020.104370 32344321 PMC7194648

[13] Tian W Jiang W Yao J Nicholson C Li R Sigurslid H Predictors of mortality in hospitalized COVID-19 patients: a systematic review and meta-analysis. *J Med Virol.* (2020) 92:1875–83. 10.1002/jmv.26050 32441789 PMC7280666

[14] Chung M Martin D Sprecher D Wazni O Kanderian A Carnes C C-reactive protein elevation in patients with atrial arrhythmias: inflammatory mechanisms and persistence of atrial fibrillation. *Circulation.* (2001) 104:2886–91. 10.1161/hc4901.101760 11739301

[15] Hu L Chen S Fu Y Gao Z Long H Ren H Risk factors associated with clinical outcomes in 323 coronavirus disease 2019 (COVID-19) hospitalized patients in Wuhan, China. *Clin Infect Dis.* (2020) 71:2089–98. 10.1093/cid/ciaa539 32361738 PMC7197620

[16] Wang D Hu B Hu C Zhu F Liu X Zhang J Clinical characteristics of 138 hospitalized patients with 2019 novel coronavirus-infected pneumonia in Wuhan, China. *JAMA.* (2020) 323:1061–9. 10.1001/jama.2020.1585 32031570 PMC7042881

[17] Bhatla A Mayer M Adusumalli S Hyman M Oh E Tierney A COVID-19 and cardiac arrhythmias. *Heart Rhythm.* (2020) 17:1439–44. 10.1016/j.hrthm.2020.06.016 32585191 PMC7307518

[18] Benjamin E Levy D Vaziri S D’Agostino R Belanger A Wolf P. Independent risk factors for atrial fibrillation in a population-based cohort. The Framingham heart study. *JAMA.* (1994) 271:840–4. 8114238

[19] Roselli C Rienstra M Ellinor P. Genetics of atrial fibrillation in 2020: GWAS, genome sequencing, polygenic risk, and beyond. *Circ Res.* (2020) 127:21–33. 32716721 10.1161/CIRCRESAHA.120.316575 PMC7388073

[20] Miyasaka Y Barnes M Gersh B Cha S Bailey K Abhayaratna W Secular trends in incidence of atrial fibrillation in Olmsted County, Minnesota, 1980 to 2000, and implications on the projections for future prevalence. *Circulation.* (2006) 114:119–25. 16818816 10.1161/CIRCULATIONAHA.105.595140

[21] Molina C Abu-Taha I Wang Q Roselló-Díez E Kamler M Nattel S Profibrotic, electrical, and calcium-handling remodeling of the atria in heart failure patients with and without atrial fibrillation. *Front Physiol.* (2018) 9:1383. 10.3389/fphys.2018.01383 30356673 PMC6189336

[22] Pluteanu F Nikonova Y Holzapfel A Herzog B Scherer A Preisenberger J Progressive impairment of atrial myocyte function during left ventricular hypertrophy and heart failure. *J Mol Cell Cardiol.* (2018) 114:253–63. 10.1016/j.yjmcc.2017.11.020 29191788

[23] Aistrup G Arora R Grubb S Yoo S Toren B Kumar M Triggered intracellular calcium waves in dog and human left atrial myocytes from normal and failing hearts. *Cardiovasc Res.* (2017) 113:1688–99. 29016724 10.1093/cvr/cvx167 PMC5852523

[24] Xu J Cui G Esmailian F Plunkett M Marelli D Ardehali A Atrial extracellular matrix remodeling and the maintenance of atrial fibrillation. *Circulation.* (2004) 109:363–8. 14732752 10.1161/01.CIR.0000109495.02213.52

[25] Ma J Chen Q Ma S. Left atrial fibrosis in atrial fibrillation: mechanisms, clinical evaluation and management. *J Cell Mol Med.* (2021) 25:2764–75. 33576189 10.1111/jcmm.16350 PMC7957273

[26] Hindricks G Potpara T Dagres N Arbelo E Bax J Blomström-Lundqvist C 2020 ESC Guidelines for the diagnosis and management of atrial fibrillation developed in collaboration with the European association for cardio-thoracic surgery (EACTS): the task force for the diagnosis and management of atrial fibrillation of the European society of cardiology (ESC) developed with the special contribution of the European heart rhythm association (EHRA) of the ESC. *Eur Heart J.* (2021) 42:373–498. 32860505 10.1093/eurheartj/ehaa612

[27] Yao C Veleva T Scott L Jr. Cao S Li L Chen G Enhanced cardiomyocyte NLRP3 inflammasome signaling promotes atrial fibrillation. *Circulation.* (2018) 138:2227–42. 29802206 10.1161/CIRCULATIONAHA.118.035202 PMC6252285

[28] Sirish P Li N Timofeyev V Zhang X Wang L Yang J Molecular mechanisms and new treatment paradigm for atrial fibrillation. *Circ Arrhythm Electrophysiol.* (2016) 9:e003721. 10.1161/CIRCEP.115.003721 27162031 PMC4869994

[29] Harhay J Khan M Shah S Malhotra A. SARS-COV-2 presenting as new onset atrial fibrillation: a case report. *Cureus.* (2020) 12:e8054. 10.7759/cureus.8054 PMC7286580 32537272

[30] Inciardi R Adamo M Lupi L Cani D Di Pasquale M Tomasoni D Characteristics and outcomes of patients hospitalized for COVID-19 and cardiac disease in Northern Italy. *Eur Heart J.* (2020) 41:1821–9. 10.1093/eurheartj/ehaa388 32383763 PMC7239204

[31] Spinoni E Mennuni M Rognoni A Grisafi L Colombo C Lio V Contribution of atrial fibrillation to in-hospital mortality in patients with COVID-19. *Circ Arrhythm Electrophysiol.* (2021) 14:e009375. 10.1161/CIRCEP.120.009375 33591815 PMC7892203

[32] Colon C Barrios J. Atrial arrhythmias in COVID-19 patients. *JACC Clin Electrophysiol.* (2021) 6:1189–90. 10.1016/j.jacep.2020.05.015 32972558 PMC7253953

[33] Musikantow D. Atrial fibrillation in patients hospitalized with COVID-19: incidence, predictors, outcomes, and comparison to influenza. *JACC Clin Electrophysiol.* (2021) 7:1120–30. 10.1016/j.jacep.2021.02.009 33895107 PMC7904279

[34] Tirandi A Ramoni D Montecucco F Liberale L. Predicting mortality in hospitalized COVID-19 patients. *Intern Emerg Med.* (2022) 17:1571–4. 10.1007/s11739-022-03017-6 35704169 PMC9198615

[35] Niedziela J Jaroszewicz J Wita K Cieśla D Ga̧sior M. High in-hospital and post-discharge mortality in patients with a pre-existing diagnosis of heart failure hospitalized due to COVID-19. *Kardiol Pol.* (2022) 80:90–2. 10.33963/KP.a2021.0163 34845713

[36] Maisano A Vitolo M Imberti J Bonini N Albini A Valenti A Atrial fibrillation in the setting of acute pneumonia: not a secondary arrhythmia. *Rev Cardiovasc Med.* (2022) 23:176. 10.31083/j.rcm2305176 39077611 PMC11273968

[37] Wang R Macha K Haupenthal D Gaßmann L Siedler G Stoll S Acute care and secondary prevention of stroke with newly detected versus known atrial fibrillation. *Eur J Neurol.* (2022) 29:1963–71. 10.1111/ene.15338 35344638

[38] Young L Antwi-Boasiako S Ferrall J Wold L Mohler P El Refaey M. Genetic and non-genetic risk factors associated with atrial fibrillation. *Life Sci.* (2022) 299:120529. 10.1016/j.lfs.2022.120529 35385795 PMC9058231

[39] El-Arif G Khazaal S Farhat A Harb J Annweiler C Wu Y Angiotensin II type I receptor (AT1R): the gate towards COVID-19-associated diseases. *Molecules.* (2022) 27:2048. 10.3390/molecules27072048 35408447 PMC9000463

[40] García-Escobar A Vera-Vera S Jurado-Román A Jiménez-Valero S Galeote G Moreno R. Calcium signaling pathway is involved in the shedding of ACE2 catalytic ectodomain: new insights for clinical and therapeutic applications of ACE2 for COVID-19. *Biomolecules.* (2022) 12:76. 10.3390/biom12010076 35053224 PMC8774087

[41] Samavati L Uhal B. ACE2, much more than just a receptor for SARS-COV-2. *Front Cell Infect Microbiol.* (2020) 10:317. 10.3389/fcimb.2020.00317 32582574 PMC7294848

[42] Avolio E Carrabba M Milligan R Kavanagh Williamson M Beltrami A Gupta K The SARS-CoV-2 Spike protein disrupts human cardiac pericytes function through CD147 receptor-mediated signalling: a potential non-infective mechanism of COVID-19 microvascular disease. *Clin Sci (Lond).* (2021) 135:2667–89. 10.1042/CS20210735 34807265 PMC8674568

[43] Hoffmann M Kleine-Weber H Schroeder S Krüger N Herrler T Erichsen S SARS-CoV-2 cell entry depends on ACE2 and TMPRSS2 and is blocked by a clinically proven protease inhibitor. *Cell.* (2020) 181:271–80.e8. 10.1016/j.cell.2020.02.052 32142651 PMC7102627

[44] Goudis C Kallergis E Vardas P. Extracellular matrix alterations in the atria: insights into the mechanisms and perpetuation of atrial fibrillation. *Europace.* (2012) 14:623–30. 10.1093/europace/eur398 22237583

[45] Rodrigues R Costa de Oliveira S. The impact of angiotensin-converting enzyme 2 (ACE2) expression levels in patients with comorbidities on COVID-19 severity: a comprehensive review. *Microorganisms.* (2021) 9:1692. 10.3390/microorganisms9081692 34442770 PMC8398209

[46] Lu Y Chen Y Kao Y Wu T Chen S Chen Y. Extracellular matrix of collagen modulates intracellular calcium handling and electrophysiological characteristics of HL-1 cardiomyocytes with activation of angiotensin II type 1 receptor. *J Card Fail.* (2011) 17:82–90. 10.1016/j.cardfail.2010.10.002 21187267

[47] Fong S Agrawal S Gong M Zhao J. Modulated calcium homeostasis and release events under atrial fibrillation and its risk factors: a meta-analysis. *Front Cardiovasc Med.* (2021) 8:662914. 10.3389/fcvm.2021.662914 34355025 PMC8329373

[48] Ferrario C Ahmad S Groban L. Mechanisms by which angiotensin-receptor blockers increase ACE2 levels. *Nat Rev Cardiol.* (2020) 17:378. 10.1038/s41569-020-0387-7 32332868 PMC7181109

[49] Blume C Jackson C Spalluto C Legebeke J Nazlamova L Conforti F A novel ACE2 isoform is expressed in human respiratory epithelia and is upregulated in response to interferons and RNA respiratory virus infection. *Nat Genet.* (2021) 53:205–14. 10.1038/s41588-020-00759-x 33432184

[50] Gheware A Ray A Rana D Bajpai P Nambirajan A Arulselvi S ACE2 protein expression in lung tissues of severe COVID-19 infection. *Sci Rep.* (2022) 12:4058. 10.1038/s41598-022-07918-6 35260724 PMC8902283

[51] Iwasaki M Saito J Zhao H Sakamoto A Hirota K Ma D. Inflammation triggered by SARS-CoV-2 and ACE2 augment drives multiple organ failure of severe COVID-19: molecular mechanisms and implications. *Inflammation.* (2021) 44:13–34. 10.1007/s10753-020-01337-3 33029758 PMC7541099

[52] Zou X Chen K Zou J Han P Hao J Han Z. Single-cell RNA-seq data analysis on the receptor ACE2 expression reveals the potential risk of different human organs vulnerable to 2019-nCoV infection. *Front Med.* (2020) 14:185–92. 10.1007/s11684-020-0754-0 32170560 PMC7088738

[53] Behl T Kaur I Aleya L Sehgal A Singh S Sharma N CD147-spike protein interaction in COVID-19: get the ball rolling with a novel receptor and therapeutic target. *Sci Total Environ.* (2021) 808:152072. 10.1016/j.scitotenv.2021.152072 34863742 PMC8634688

[54] Wang K Chen W Zhang Z Deng Y Lian J Du P CD147-spike protein is a novel route for SARS-CoV-2 infection to host cells. *Signal Transduct Target Ther.* (2020) 5:283. 10.1038/s41392-020-00426-x 33277466 PMC7714896

[55] Fenizia C Galbiati S Vanetti C Vago R Clerici M Tacchetti C SARS-CoV-2 entry: at the crossroads of CD147 and ACE2. *Cells.* (2021) 10:1434. 10.3390/cells10061434 34201214 PMC8226513

[56] Mahdian S Zarrabi M Panahi Y Dabbagh S. Repurposing FDA-approved drugs to fight COVID-19 using in silico methods: targeting SARS-CoV-2 RdRp enzyme and host cell receptors (ACE2, CD147) through virtual screening and molecular dynamic simulations. *Inform Med Unlocked.* (2021) 23:100541. 10.1016/j.imu.2021.100541 33649734 PMC7904474

[57] Peng X Wang Y Xi X Jia Y Tian J Yu B Promising therapy for heart failure in patients with severe COVID-19: calming the cytokine storm. *Cardiovasc Drugs Ther.* (2021) 35:231–47. 10.1007/s10557-020-07120-8 33404925 PMC7786163

[58] Pituch-Noworolska A. NK cells in SARS-CoV-2 infection. *Cent Eur J Immunol.* (2022) 47:95–101. 10.5114/ceji.2022.113078 35600151 PMC9115590

[59] Li X Guo X Chang Y Zhang N Sun Y. Analysis of alterations of serum inflammatory cytokines and fibrosis makers in patients with essential hypertension and left ventricular hypertrophy and the risk factors. *Am J Transl Res.* (2022) 14:4097–103. 35836904 PMC9274558

[60] Ueland T Holter J Holten A Müller K Lind A Bekken G Distinct and early increase in circulating MMP-9 in COVID-19 patients with respiratory failure. *J Infect.* (2020) 81:e41–3. 10.1016/j.jinf.2020.06.061 32603675 PMC7320854

[61] Gelzo M Cacciapuoti S Pinchera B Rosa AD Cernera G Scialò F Matrix metalloproteinases (MMP) 3 and 9 as biomarkers of severity in COVID-19 patients. *Sci Rep.* (2022) 12:1212. 10.1038/s41598-021-04677-8 35075175 PMC8786927

[62] Savic G Stevanovic I Mihajlovic D Jurisevic M Gajovic N Jovanovic I MMP-9/BDNF ratio predicts more severe COVID-19 outcomes. *Int J Med Sci.* (2022) 19:1903–11. 10.7150/ijms.75337 36438922 PMC9682503

[63] Matrosovich M Herrler G Klenk H. Sialic acid receptors of viruses. *Top Curr Chem.* (2015) 367:1–28. 10.1007/128_2013_466 23873408 PMC7120183

[64] Hu W Xie J Zhu T Meng G Wang M Zhou Z Serum N-acetylneuraminic acid is associated with atrial fibrillation and left atrial enlargement. *Cardiol Res Pract.* (2020) 2020:1358098. 10.1155/2020/1358098 32351730 PMC7174944

[65] Kochi A Tagliari A Forleo G Fassini G Tondo C. Cardiac and arrhythmic complications in patients with COVID-19. *J Cardiovasc Electrophysiol.* (2020) 31:1003–8. 10.1111/jce.14479 32270559 PMC7262150

[66] Clementy N Piver E Bisson A Andre C Bernard A Pierre B Galectin-3 in atrial fibrillation: mechanisms and therapeutic implications. *Int J Mol Sci.* (2018) 19:976. 10.3390/ijms19040976 29587379 PMC5979515

[67] Cannavo A Liccardo D Gelzo M Amato F Gentile I Pinchera B Serum galectin-3 and aldosterone: potential biomarkers of cardiac complications in patients with COVID-19. *Minerva Endocrinol.* (2022) 47:270–8. 10.23736/S2724-6507.22.03789-7 35266671

[68] Cervantes-Alvarez E la Rosa N la Mora M Valdez-Sandoval P Palacios-Jimenez M Rodriguez-Alvarez F Galectin-3 as a potential prognostic biomarker of severe COVID-19 in SARS-CoV-2 infected patients. *Sci Rep.* (2022) 12:1856. 10.1038/s41598-022-05968-4 35115644 PMC8813958

[69] Tang D Comish P Kang R. The hallmarks of COVID-19 disease. *PLoS Pathog.* (2020) 16:e1008536. 10.1371/journal.ppat.1008536 32442210 PMC7244094

[70] Farahani M Niknam Z Mohammadi Amirabad L Amiri-Dashatan N Koushki M Nemati M Molecular pathways involved in COVID-19 and potential pathway-based therapeutic targets. *Biomed Pharmacother.* (2022) 145:112420. 10.1016/j.biopha.2021.112420 34801852 PMC8585639

[71] Aleebrahim-Dehkordi E Molavi B Mokhtari M Deravi N Fathi M Fazel T T helper type (Th1/Th2) responses to SARS-CoV-2 and influenza A (H1N1) virus: from cytokines produced to immune responses. *Transpl Immunol.* (2022) 70:101495. 10.1016/j.trim.2021.101495 34774738 PMC8579696

[72] Mazzoni A Salvati L Maggi L Annunziato F Cosmi L. Hallmarks of immune response in COVID-19: exploring dysregulation and exhaustion. *Semin Immunol.* (2021) 55:101508. 10.1016/j.smim.2021.101508 34728121 PMC8547971

[73] Li S Wang J Yan Y Zhang Z Gong W Nie S. Clinical characterization and possible pathological mechanism of acute myocardial injury in COVID-19. *Front Cardiovasc Med.* (2022) 9:862571. 10.3389/fcvm.2022.862571 35387441 PMC8979292

[74] Shukla A Misra S. Antimicrobials in COVID-19: strategies for treating a COVID-19 pandemic. *J Basic Clin Physiol Pharmacol.* (2022). 10.1515/jbcpp-2022-0061 35503307

[75] Che Mohd Nassir C Zolkefley M Ramli M Norman H Abdul Hamid H Mustapha M. Neuroinflammation and COVID-19 ischemic stroke recovery-evolving evidence for the mediating roles of the ACE2/angiotensin-(1-7)/Mas receptor axis and NLRP3 inflammasome. *Int J Mol Sci.* (2022) 23:3085. 10.3390/ijms23063085 35328506 PMC8949282

[76] Rodrigues T de Sá K Ishimoto A Becerra A Oliveira S Almeida L Inflammasomes are activated in response to SARS-CoV-2 infection and are associated with COVID-19 severity in patients. *J Exp Med.* (2021) 218:e20201707. 10.1084/jem.20201707 33231615 PMC7684031

[77] Caporali S De Stefano A Calabrese C Giovannelli A Pieri M Savini I Anti-inflammatory and active biological properties of the plant-derived bioactive compounds luteolin and luteolin 7-glucoside. *Nutrients.* (2022) 14:1155. 10.3390/nu14061155 35334812 PMC8949538

[78] Saljic A Heijman J Dobrev D. Emerging antiarrhythmic drugs for atrial fibrillation. *Int J Mol Sci.* (2022) 23:4096. 10.3390/ijms23084096 35456912 PMC9029767

[79] Ruhl L Pink I Kühne J Beushausen K Keil J Christoph S Endothelial dysfunction contributes to severe COVID-19 in combination with dysregulated lymphocyte responses and cytokine networks. *Signal Transduct Target Ther.* (2021) 6:418. 10.1038/s41392-021-00819-6 34893580 PMC8661333

[80] Nägele M Haubner B Tanner F Ruschitzka F Flammer A. Endothelial dysfunction in COVID-19: current findings and therapeutic implications. *Atherosclerosis.* (2020) 314:58–62. 10.1016/j.atherosclerosis.2020.10.014 33161318 PMC7554490

[81] Otifi H Adiga B. Endothelial dysfunction in covid-19 infection. *Am J Med Sci.* (2022) 363:281–7. 10.1016/j.amjms.2021.12.010 35093394 PMC8802031

[82] Cooper S Boyle E Jefferson S Heslop C Mohan P Mohanraj G Role of the renin-angiotensin-aldosterone and Kinin-Kallikrein systems in the cardiovascular complications of COVID-19 and long COVID. *Int J Mol Sci.* (2021) 22:8255. 10.3390/ijms22158255 34361021 PMC8347967

[83] Bittner Z Schrader M George S Amann R. Pyroptosis and its role in SARS-CoV-2 infection. *Cells.* (2022) 11:1717. 10.3390/cells11101717 35626754 PMC9140030

[84] Chen A Wang C Zhu W Chen W. Coagulation disorders and thrombosis in COVID-19 patients and a possible mechanism involving endothelial cells: a review. *Aging Dis.* (2022) 13:144–56. 10.14336/AD.2021.0704 35111367 PMC8782553

[85] Fischer L Barop H Ludin S Schaible H. Regulation of acute reflectory hyperinflammation in viral and other diseases by means of stellate ganglion block. A conceptual view with a focus on covid-19. *Auton Neurosci.* (2022) 237:102903. 10.1016/j.autneu.2021.102903 34894589 PMC9761017

[86] Stute N Stickford J Province V Augenreich M Ratchford S Stickford A. COVID-19 is getting on our nerves: sympathetic neural activity and haemodynamics in young adults recovering from SARS-CoV-2. *J Physiol.* (2021) 599:4269–85. 10.1113/JP281888 34174086 PMC8447023

[87] Bers D. Cardiac excitation-contraction coupling. *Nature.* (2022) 415:198–205. 10.1038/415198a 11805843

[88] Raatikainen P Lassila R. COVID-19: another reason for anticoagulation in patients with atrial fibrillation. *Heart.* (2022) 108:902–4. 10.1136/heartjnl-2022-320845 35314451

[89] Gawałko M Kapłon-Cieślicka A Hohl M Dobrev D Linz D. COVID-19 associated atrial fibrillation: incidence, putative mechanisms and potential clinical implications. *Int J Cardiol Heart Vasc.* (2020) 30:100631. 10.1016/j.ijcha.2020.100631 32904969 PMC7462635

[90] Qin W Dong F Zhang Z Hu B Chen S Zhu Z Low molecular weight heparin and 28-day mortality among patients with coronavirus disease 2019: a cohort study in the early epidemic era. *Thromb Res.* (2021) 198:19–22. 10.1016/j.thromres.2020.11.020 33249247 PMC7681071

[91] Russo V Rago A Carbone A Bottino R Ammendola E Della Cioppa N Atrial fibrillation in COVID-19: from epidemiological association to pharmacological implications. *J Cardiovasc Pharmacol.* (2020) 76:138–45. 10.1097/FJC.0000000000000854 32453074

[92] Gronich N Stein N Muszkat M. Association between use of pharmacokinetic-interacting drugs and effectiveness and safety of direct acting oral anticoagulants: nested case-control study. *Clin Pharmacol Ther.* (2021) 110:1526–36. 10.1002/cpt.2369 34287842 PMC9290518

[93] Lindahl U Li J. Heparin–an old drug with multiple potential targets in covid-19 therapy. *J Thromb Haemost.* (2020) 18:2422–4. 10.1111/jth.14898 32426897 PMC7276884

[94] Harrison S Fazio-Eynullayeva E Lane D Underhill P Lip G. Atrial fibrillation and the risk of 30-day incident thromboembolic events, and mortality in adults ≥ 50 years with COVID-19. *J Arrhythm.* (2020) 37:231–7. 10.1002/joa3.12458 33664908 PMC7896479

[95] Shah D Umar Z Ilyas U Nso N Zirkiyeva M Rizzo V. New-onset atrial fibrillation in COVID-19 infection: a case report and review of literature. *Cureus.* (2022) 14:e23912. 10.7759/cureus.23912 35530910 PMC9076057

[96] Marcianò G Roberti R Palleria C Mirra D Rania V Casarella A SARS-CoV-2 treatment: current therapeutic options and the pursuit of tailored therapy. *Appl Sci.* (2021) 11:7457. 10.3390/app11167457

[97] Karamchandani K Quintili A Landis T Bose S. Cardiac arrhythmias in critically Ill patients with COVID-19: a brief review. *J Cardiothorac Vasc Anesth.* (2021) 35:3789–96. 10.1053/j.jvca.2020.08.013 32888796 PMC7418708

[98] REMAP-CAP Investigators, ACTIV-4a Investigators, ATTACC Investigators Goligher E Bradbury C McVerry B Therapeutic anticoagulation with heparin in critically ill patients with covid-19. *N Engl J Med.* (2021) 385:777–89. 10.1056/NEJMoa2103417 34351722 PMC8362592

[99] Gasperetti A Schiavone M Tondo C Mitacchione G Viecca M Galli M QT interval monitoring and drugs management during COVID-19 pandemic. *Curr Rev Clin Exp Pharmacol.* (2021) 16:306–17. 10.2174/1574884715666201224155042 33357185

[100] Wu C Postema P Arbelo E Behr E Bezzina C Napolitano C SARS-CoV-2, COVID-19, and inherited arrhythmia syndromes. *Heart Rhythm.* (2020) 17:1456–62. 10.1016/j.hrthm.2020.03.024 32244059 PMC7156157

[101] Fresse A Viard D Romani S Gérard A Lepelley M Rocher F Spontaneous reported cardiotoxicity induced by lopinavir/ritonavir in COVID-19. An alleged past-resolved problem. *Int J Cardiol.* (2021) 324:255–60. 10.1016/j.ijcard.2020.10.028 33075384 PMC7566676

[102] Thibaud S Tremblay D Bhalla S Zimmerman B Sigel K Gabrilove J. Protective role of Bruton tyrosine kinase inhibitors in patients with chronic lymphocytic leukaemia and COVID-19. *Br J Haematol.* (2020) 190:e73–6. 10.1111/bjh.16863 32433778 PMC7276870

[103] Treon S Castillo J Skarbnik A Soumerai J Ghobrial I Guerrera M. The BTK inhibitor ibrutinib may protect against pulmonary injury in COVID-19-infected patients. *Blood.* (2020) 135:1912–5. 10.1182/blood.2020006288 32302379 PMC7243149

[104] Salem J Manouchehri A Bretagne M Lebrun-Vignes B Groarke J Johnson D Cardiovascular toxicities associated with ibrutinib. *J Am Coll Cardiol.* (2019) 74:1667–78. 10.1016/j.jacc.2019.07.056 31558250

[105] Coleman C Sisk J Mingo R Nelson E White J Frieman M. Abelson kinase inhibitors are potent inhibitors of severe acute respiratory syndrome coronavirus and Middle East respiratory syndrome coronavirus fusion. *J Virol.* (2016) 90:8924–33. 10.1128/jvi.01429-16 27466418 PMC5021412

[106] Hartmann J Haap M Kopp H Lipp H. Tyrosine kinase inhibitors–a review on pharmacology, metabolism and side effects. *Curr Drug Metab.* (2009) 10:470–81. 10.2174/138920009788897975 19689244

[107] Malin J Suárez I Priesner V Fätkenheuer G Rybniker J. Remdesivir against COVID-19 and other viral diseases. *Clin Microbiol Rev.* (2020) 34:e162–120. 10.1128/CMR.00162-20 33055231 PMC7566896

[108] Grein J Ohmagari N Shin D Diaz G Asperges E Castagna A Compassionate use of Remdesivir for patients with severe covid-19. *N Engl J Med.* (2020) 382:2327–36. 10.1056/NEJMoa2007016 32275812 PMC7169476

[109] Michaud V Dow P Al Rihani S Deodhar M Arwood M Cicali B Risk assessment of drug-induced long QT syndrome for some COVID-19 repurposed drugs. *Clin Transl Sci.* (2021) 14:20–8. 10.1111/cts.12882 32888379 PMC7877829

[110] Rafaniello C Ferrajolo C Sullo M Gaio M Zinzi A Scavone C Cardiac events potentially associated to Remdesivir: an analysis from the European spontaneous adverse event reporting system. *Pharmaceuticals (Basel).* (2021) 14:611. 10.3390/ph14070611 34202350 PMC8308754

[111] Bistrovic P Lucijanic M. Remdesivir might induce changes in electrocardiogram beyond bradycardia in patients with coronavirus disease 2019-the pilot study. *J Med Virol.* (2021) 93:5724–5. 10.1002/jmv.27177 34232520 PMC8426664

